# Study of collaborative filtering recommendation with user clustering incorporating implicit social relationships and trust relationships

**DOI:** 10.1371/journal.pone.0332998

**Published:** 2025-10-06

**Authors:** Yan Li, Xue Lin

**Affiliations:** School of Economics and Management, Shanghai Maritime University, Shanghai, China; Education University of Hong Kong, CHINA

## Abstract

With the rapid development of the internet, information overload has become a prevalent issue. In order to tackle information overload, recommendation systems serve as an effective tool that can offer personalized recommendation services to users. The efficiency of recommendation systems is, however, hampered by the prevalent problems with data sparsity and cold start issues in collaborative filtering recommendations. Researchers typically address these issues by utilizing user social information clustering methods. Nevertheless, in practice, previous studies have shown that inaccurate similarity calculations and poor clustering results have led to a decrease in prediction accuracy. This paper suggests a collaborative filtering recommendation algorithm that incorporates several relationships in order to overcome these difficulties. This method first calculates user similarity based on implicit social relationships and trust relationships. After clustering users using the spectral clustering technique, it makes use of user-based collaborative filtering recommendations within the cluster containing the target person. The collaborative filtering recommendation system that integrates many relationships effectively decreases prediction errors and improves recommendation accuracy, as shown by the results of simulated studies.

## Introduction

The proliferation of the Internet has led to the widespread issue of information overload [1]. Recommender systems, as one of the effective tools to cope with information overload, provide users with personalized recommendation services. However, in emerging online retail platforms like newly established online bookstores, two typical phenomena can be observed: the cold start problem and data sparsity issue. First, as the platform has just been launched, the number of newly registered users is limited, and many users have not yet performed activities such as purchasing or rating on the platform, which is a manifestation of the cold start problem. The lack of behavioral data from these users makes it difficult for the recommender system to accurately recommend books for them that match their interests. Second, even if some users have already made purchases and ratings on the platform, due to the wide variety of books, many books have only been purchased or rated by a few users, resulting in very sparse data on the interaction between users and books. For example, some academic books in specific fields may only be purchased by a few users, and most users do not have any behavioral data, which is a data sparsity problem. The recommender system finds it challenging to determine the user’s preference for these uncommon books in this instance, which has an impact on the recommendation’s accuracy.

Collaborative filtering (CF), a popular and significant recommendation technique in the field of recommender systems [2], is predicated on the idea of making recommendations by examining user or item similarity[3,4] Collaborative filtering is able to dynamically adjust the recommendation results according to the user’s behavior to adjust to the user’s shifting interests [5]. However, in practical applications, collaborative filtering methods often face data sparsity and cold-start problems. To address these problems, researchers have proposed new perspectives based on user similarity and trust [6–9] and used hierarchical clustering [6], matrix factorization [7], and user clustering [8,9] to improve recommendation accuracy. These new methods successfully enhance recommendation systems’ efficacy by better exploring the similarities and correlations between users.

According to previous studies, clustering-based collaborative filtering methods have been shown to be one of the effective means to address the issue of cold-starting. Meanwhile, the sparsity of data can be effectively reduced by calculating the similarity between users. It is found that user clustering is mainly based on explicit or implicit relationships between users, however, there are still the following aspects that need to be further explored and solved:

1. Some social network-based recommendation studies require explicit social relationships of users; however, not all recommender systems have access to explicit social information. Meanwhile, explicit information about items is often scarce, so implicit information is needed to improve recommendation accuracy. Therefore, one of the difficulties facing recommendation algorithm research is figuring out how to effectively use implicit interaction information to boost recommender system accuracy.

2. Current recommendation research is mainly based on existing rating data to analyze user and item information, and use this information to calculate user similarity and perform user clustering. However, these studies often neglect the trust relationship between users, resulting in large errors in the user similarity computation and a poor clustering effect, which affects the prediction accuracy. In fact, there are differences between social and trust relationships. Social relationships refer to the connectivity of user behaviors based on shared interests, measuring the degree of interest overlap between users, primarily using the user-item rating matrix and friendship networks. In contrast, trust relationships involve the subjective assessment of the credibility of others’ ratings, evaluating the reliability of rating behaviors, mainly based on the consistency of ratings for items that are commonly rated. Social relationships reflect the breadth of interest associations, while trust relationships embody the depth of preference consistency. A well-established trust relationship model can help to find more similar nearest neighbors and increase the recommendation system’s accuracy. Therefore, how to accurately calculate the similarity between users in order to group people with related interests together is an urgent topic for further exploration in the field of RS.

Although studies have been conducted on these issues, a deeper and comprehensive exploration of these related issues has not yet been conducted. Therefore, the purpose of this research is to develop a user clustering collaborative filtering recommendation algorithm that takes implicit social and trust relationships into account to overcome the aforementioned difficulties. Overall, the main elements of this study are:

1. In this study, we construct user similarity using a trust relationship model based on the intersection of other users’ ratings of target users’ rated items and the size of the ratings, and we use the improved Jaccard similarity coefficient to calculate the strength of social relationships between users. This model integrates the connections between users by linearly fusing social and trust relationships to produce a final user similarity.
2. Within the target user group in this study, we employ a user-based collaborative filtering recommendation method after grouping people using a spectral clustering algorithm.
3. Experimental results on the MovieLens-100k dataset show that this approach performs well and effectively improves recommendation accuracy compared to previous schemes, while effectively mitigating the cold-start and data sparsity problems.

## Related work

Considering the problem studied and the methodology used in this study, a research review on the following two topics, including CF algorithms and user relationship-based recommendation algorithms, is presented.

### Collaborative filtering recommendation algorithm

Previous research on CF algorithms can be divided into two categories: memory-based CF algorithms and model-based CF algorithms [10]. Memory-based CF algorithms include user-based CF (UserCF) and item-based CF (ItemCF), which are mainly based on user behavioral data or similarity between items to make recommendations. The algorithm is simple and intuitive, based on the user’s rating data or behavioral data of the item, the user-item rating matrix is constructed [4,11,12], and the similarity metric calculates the distance between them based on the constructed rating matrix, so the algorithm is incapable of handling the cold-start issue and is sensitive to data sparsity.

Model-based CF methods are used to solve these problems, which learn the potential features of users and items from user-item interaction data by building machine learning models, and then make recommendations. Commonly used models include Singular Value Decomposition (SVD) [13–15], Hidden Semantic Models (LFM) [16], Factorial Decomposition Machines (FMs) [17], and Clustered Recommendation Models [2,9,18–20], etc. Model-based techniques outperform memory-based techniques in handling data sparsity, cold-start issues, and providing superior recommendation outcomes and scalability.

Among the model-based collaborative filtering methods, clustering recommendation model is widely used, which is a clustering algorithm that clusters users or items with similar attributes together by calculating the similarity of the users or items. Refer to the literature [21], where the authors suggest a collaborative recommender that groups comparable users using the K-means clustering method and predicts the user’s rating for a particular item using a user-based model. Literature [8,18] studies concluded that FCM outperforms K-means. The algorithm proposed in literature [18] transforms user preferences for individual items into similar clusters through fuzzy C-means clustering and establishes sparse user-item preferences as dense user-fuzzy preferences. Finally, it finds the target user’s nearest neighbors based on item clustering and generates a recommendation. Literature [19] introduced information entropy and bi-clustering into CF, and proposed a novel collaborative filtering method (CBE-CF) to extract locally dense scoring modules to address the issues with data sparsity and computational efficiency that come with traditional recommendation algorithms. However, these methods also have some problems. Determining the right number of clusters is a major issue in certain clustering techniques (such as fuzzy C-means and K-means), as the effectiveness of the method depends on a predetermined ideal number of clusters. The identified clusters’ usefulness is another problem. Poorer clustering results may lead to lower prediction accuracy and coverage of recommendation results.

According to the research results of literature [22], the spectral clustering algorithm can effectively utilize the strength of the relationship between users in their social relationships and achieve good clustering results. This study constructed a user relationship model in the following steps, and the spectral clustering algorithm is used for user clustering in order to obtain better clustering results and to divide users more reasonably.

### Recommendation algorithms based on user relationships

An important area of research in recommender systems is user relationship-based recommendation algorithms, which can enhance the performance and user experience of recommender systems by mining the social relationship information between users. User relationship-based recommendation algorithm is a method that utilizes the social relationship data between users to enhance recommendation system performance. This algorithm analyzes social network relationships between users, such as friend relationships and following relationships, to recommend items that users may be interested in. By mining the social relationships between users, the interests and behaviors of users can be better understood and the accuracy of recommendations can be improved.

In user relationship-based recommendation algorithms, commonly used methods include social network-based recommendation [23,24], social influence-based recommendation [8,13,25,26], and so on. These methods provide users with appropriate recommendation items by analyzing factors such as relationships between users, interaction behaviors, and influence. In the field of recommender systems and social networks, user relationships can be categorized into explicit user relationships and implicit user relationships. Explicit user relationship refers to the explicitly expressed or confirmed relationship between users, such as friend relationship, family relationship, etc. This kind of relationship is usually established or chosen by the users themselves, and has a clear social connection. Implicit user relationship refers to the relationship that exists between users but is not explicitly expressed or confirmed, such as similar behaviors, common visits to websites, etc., and this relationship is usually derived through user’s behavior or data analysis.

Social recommendation requires the collection of each user’s social relationships to build a social graph, which poses the risk of violating users’ privacy due to untrustworthy servers [27,28], and it is difficult for researchers to have direct access to users’ explicit relationships. Literature [29,30] proposes collaborative filtering algorithms based on social networks, which can enhance the accuracy of recommendations to some degree, but require explicit social relationships between users, however, this information is not available in some systems. Therefore, existing research has increasingly emphasized on the implicit information of users. To predict user preferences for groups in a unified framework, literature [23] proposed the Knowledge Enhanced Trust Propagation Graph Neural Network (KTPGN), which takes into account two aspects of trustworthiness to construct an implicit social trust graph. It does this by combining social trust, multiple group attributes, and user-group interaction history. The methodology put forth in the literature [24] combines rating and trust information through the use of attention behavior information, and the model explicitly models users’ attention and preference for social recommendations without ignoring their implicit information. Literature [31] proposes an implicit trust network construction method that consists of two parts, i.e., constructing an initial trust network by examining users’ similarity, confidence, and agreement, and reconstructing the initial trust network into a final trust network by evaluating the prediction accuracy of the initial trust network with respect to unknown item ratings.

Considering that explicit relationships between users are not easily accessible, this study focuses on mining users’ implicit information. This study models users’ social and trust relationships from their history, with an emphasis on maximizing the utilization of relationship information between users, resolving issues with data sparsity and cold starts, and enhancing recommender system accuracy.

## Collaborative filtering recommendation method for user clustering based on users’ implicit social and trust relationships

In this section, we will introduce the method proposed in this study, namely, the user clustering collaborative filtering recommendation method based on users’ implicit social and trust relationships (STUBCF). The section is structured as follows: first, the study’s recommendation framework is presented; next, users’ social and trust relationships are modeled and users are grouped into different clusters; and lastly, a detailed description of the implementation of the collaborative filtering recommendations is provided.

### Collaborative filtering based recommendation framework

**Fig 1:**
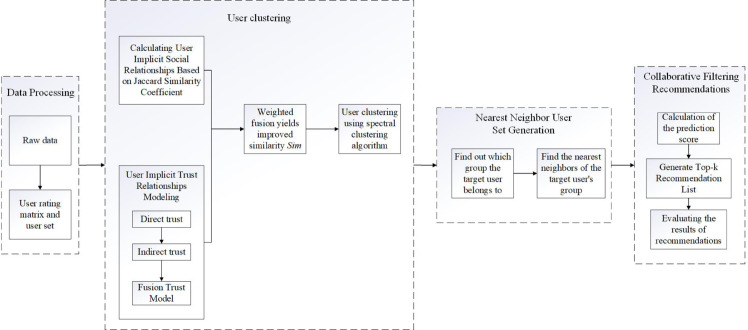
A collaborative filtering recommendation framework for user clustering that incorporates user implicit social relationships and trust relationships. A collaborative filtering recommendation framework for user clustering that incorporates user implicit social relationships and trust relationships.

The collaborative filtering recommendation process includes the steps of user behavior data collection, similarity calculation, nearest neighbor selection, recommendation result generation and evaluation metrics. The specific structure of the collaborative filtering recommendation framework for user clustering that this study proposes integrating user social relations and trust relations is depicted in Fig 1. The framework is mainly divided into four modules:

- Data Processing Module: used to perform data processing on the original dataset to generate the user-item rating matrix;
- User Clustering Module: including social relationship modeling based on the rating information, user trust modeling, and ultimately user clustering based on user similarity, which aims to classify the users;
- Nearest-neighbor Generation Module: firstly, identify the cluster to which the target user belongs, and then based on similarity, find the nearest neighbor users that are most similar to the target user within the cluster to which the target user belongs, and generate a set of nearest neighbor users;
- CF Module: based on the nearest neighbor users to compute the predicted scores of the target user and generate the Top-k recommendation list, and finally evaluate the recommendation results.

In the user clustering module, the following two improvements are made to address the problem that the sparse rating matrix causes the calculated similarity to be inaccurate and thus affects the accuracy of the final recommendation:

1. The Jaccard similarity coefficients are used to calculate the user similarity when constructing the implicit social relationship model. The improved method not only considers whether there are common rated items among users, but also considers whether there is intersection with other users who have common rated items when there are no common rated items among users, in order to more comprehensively and accurately reflect the possible interest relationships among users, and greatly enhance the user similarity’s accuracy.
2. Since the rating matrix is usually sparse with less direct trust data, the concept of indirect trust is introduced. Even if the users are not directly related to other users or the direct relation is not high, the trust is increased through the relationship established by the third party with other users.

### Similarity calculation by incorporating users’ implicit social relationships and trust relationships

#### User implicit social relationship modeling.

According to the above, this study categorizes user relationships into the following three types based on users’ implicit information (data on users’ ratings of items): ‘friends’, ‘indirect friends’, and ‘unrelated users’. Specifically, the common interest in the project between users is considered as a social relationship, and the strength of the relationship is measured using the similarity degree, which is calculated by integrating the direct interest relationship and the potential interest relationship.

The first is the “friends” relationship: this refers to users who share a common rating item. Users with common ratings are categorized as having a direct association based on similarity in their behavior on common ratings, indicating a direct sharing of interests. The formation of such social relationships is influenced by the similarity of individuals in their rating behaviors. Therefore, when different users rate the same item, we can assume that there is a similarity in their interests and therefore recognize them as “friends”.

The second is the “indirect friends” relationship: this refers to users who do not share a common rating program but have a common friend. In other words, even though there is no direct intersection of ratings between these two users, an indirect social connection is formed through their connection to another common friend. This indirect relationship shows the structure of their social network and highlights the potential interest associations generated by the common social connection between the users. Therefore, these two users are referred to as “indirect friends”.

Finally, there is the “unrelated users” relationship: users who have neither a common rating program nor a common friend.

In summary, this study improves the Jaccard similarity coefficient so that its value range is controlled between 0 and 1:

$$\text{sim}(i,j)=\left\{ \begin{array}{c} \frac{1}{1+e^{-\frac{\left|I_{i}\cap I_{j}\right|}{\left|I_{i}\cup I_{j}\right|}}},\left|I_{i}\cap I_{j}\right|!=0\\ \frac{\left|N_{i}\cap N_{j}\right|}{\left|N_{i}\cup N_{j}\right|},\left|I_{i}\cap I_{j}\right|=0\&\left|N_{i}\cap N_{j}\right|!=0\\ 0,\left|I_{i}\cap I_{j}\right|=0\&\left|N_{i}\cap N_{j}\right|=0 \end{array}\right.$$ (1)

#### User implicit trust relationship modeling.

In social relationships, each individual constructs his or her own social network, however, these relationships exist with varying degrees of closeness and distance. Although the implicit social relationships described only indicates the existence of intersections between two individuals, they do not address the level of trust between them. Therefore, this study modeled the users’ trust relationships.

Most of the current recommendation studies only consider whether there is a trust relationship between users, generally labeling trusted relationships as 1 and untrusted relationships as 0. This metric is relatively simple and fails to adequately take into account the intricacies of the degree of trust between users. Because of this, the trust relationship between users was further split into direct and indirect trust in this study. For the definition of direct trust relationship, this study draws on the concept of the TSTGR model [32], which argues that when there are common rating items and consistent ratings between users, it can be defined as the existence of a direct trust relationship. In real life, there may be a direct trust relationship between users, which is known as a direct intersection relationship, but sometimes the direct relationship between two users may not be very close. By getting to know a third user together, the relationship between these two users can be strengthened, for example, the two originally only met once, but gradually transformed into a more intimate relationship through the third friend. Therefore, indirect trustworthiness can be obtained by multiplying the direct trustworthiness of two users with that of a third user. However, if the value of a direct trust is greater than that of an indirect trust, a direct trust should be adopted. Therefore, the weighted sum of direct and indirect trust (referred to as global trust in this study) can be used to calculate the trust relationship between users.

Definition 1 (Direct trust). Direct trust between users is defined as follows:

$$DTrust\left(i,j\right)=\frac{\sum_{N_{ij}}f\left(u_{i},u_{j}\right)}{N_{ij}}$$ (2)

*N*_*ij*_ denotes the number of items jointly evaluated by users *u*_*i*_ and *u*_*j*_, and $f(u_{i},u_{j})$ denotes the rating value of the items jointly evaluated by users *u*_*i*_ and *u*_*j*_. If they agree, $f(u_{i},u_{j})=1$; otherwise, $f(u_{i},u_{j})=0$. Suppose the rating scale is r={1,2,3,4,5}. For instance, user *u*_*i*_ rates the item as 5 and user *u*_*j*_ rates the item as 4. Set $f(u_{i},u_{j})=1$ if both users’ ratings are greater than 3 (positive preference), or both users’ ratings are less than or equal to 3 (neutral preference/negative preference); otherwise set $f(u_{i},u_{j})=0$.

Definition 2 (Indirect trust). Based on the above description and the formula for direct trust, indirect trust is defined as follows:

ITrust(i,j)=max(DTrust(i,j),DTrust(i,u)×DTrust(u,j)) (3)

For example, suppose user *i* and user *j* have a direct trust relationship with a direct trust level of 0.53; user *i* and user *u* have a direct trust level of 0.56; and user *u* and user *j* have a direct trust level of 0.95. The indirect trust level between users *i* and *j* in this instance is 0.532 $\left(\begin{array}{c} 0.56\times 0.95=0.532 \end{array}\right)$. This implies that user *u* has a higher level of trust in user *j*, which affects the level of trust of user *i*’s trust in user *j*. If the calculated indirect trust is lower than the direct trust, the indirect trust will take the value of the direct trust because a person will not easily trust a person with less trust.

Definition 3 (Global Trust). Based on the direct and indirect trust built between users, this study obtains the final global trust, which is realized through the fusion of weighting factors. The value range of the weighting factor *λ* is set between 0 and 1, which is used to quantify the weights of direct and indirect trust. The specific definitions are as follows:

$$\lambda =\frac{DTrust\left(i,j\right)}{DTrust\left(i,j\right)+ITrust\left(i,j\right)}$$ (4)

The final global trust can eventually be obtained as follows:

$$Trust(\begin{array}{c} i,j \end{array})=\lambda DTrust(\begin{array}{c} i,j \end{array})+(\begin{array}{c} 1-\lambda \end{array})ITrust(\begin{array}{c} i,j \end{array})$$ (5)

#### User clustering based on spectral clustering algorithm incorporating implicit social relationships and trust relationships.

Combining the improved Jaccard similarity coefficient based on the social relationship and the user’s trust relationship, the final improved formula for measuring the similarity between users is obtained as follows:

$$Sim(\begin{array}{c} i,j \end{array})=\mu Trust(\begin{array}{c} i,j \end{array})+(\begin{array}{c} 1-\mu \end{array})sim(\begin{array}{c} i,j \end{array})$$ (6)

Spectral clustering algorithms are widely used nowadays when clustering or grouping users. Research results show that spectral clustering performs well in user clustering, especially when clustering users in social networks based on their social information. Therefore, this study finally chooses to adopt the spectral clustering algorithm to classify users into different clusters based on the user similarity derived above.

### Implementation of collaborative filtering recommendation algorithm

According to the ideas described in the previous chapter, CF is implemented in each of the obtained clusters, and the k nearest neighbor users who have the most similar users are found through the cluster where the target user is located, and the target user’s ratings are predicted using the ratings of these k users, which are computed as shown in Eq 7.

$$P_{u,i}={\overset{―}{r}}_{u}+\frac{\sum_{\nu \in l_{u}}Sim\left(u,\nu \right)\times \left(r_{\nu ,i}-{\overset{―}{r}}_{\nu }\right)}{\sum_{\nu \in l_{u}}\left|Sim\left(u,\nu \right)\right|}$$ (7)

where *P*_*u*,*i*_ denotes the predicted rating of user *u* for item *i*; $l_{u}$ is the collection of nearest neighbors searched by user *u* within its cluster; Sim(u,v) denotes the similarity between user *u* and user *v* computed and matched in Eq 6; $r_{v,i}$ represents user *v*’s actual rating for item *i*; and ${\bar{r}}_{v}$ is the average rating of user *v* over all items.

## Simulation experiments

### Experimental dataset and environment

In order to test the efficacy of the group recommendation strategy suggested in this study, the MovieLens 100K dataset, which is the most famous in the field of recommender systems, was chosen for this study. This dataset contains 100,000 rating records of 1682 movies by 943 users on a scale of 1 to 5 points. The sparsity of the dataset was calculated to be 93.7%.

The experimental environment for this study is Windows 11 platform, 64-bit operating system with 13th Gen Intel (R) Core (TM) i5-13450HX processor with 2.40 GHz and 15.7 GB of physical memory. The proposed algorithm is implemented in this study using Python 3.9.

### Evaluation metrics

Mean Absolute Error (MAE) is used as a performance indicator in the experiment to evaluate the accuracy of the methodology proposed in this study and on the prediction results.

MAE is used to calculate the error that exists between the predicted and actual values; a lower value of MAE indicates a smaller error and a higher recommendation accuracy. Its calculation formula is:

$$MAE=\frac{\sum_{i=1}^{N}\left|P_{i}-R_{i}\right|}{N}$$ (8)

where *P*_*i*_ represents the target user’s predicted rating of item *i* as generated by the algorithm, *R*_*i*_ represents the target user’s actual rating of item *i*, and *N* denotes the number of items that have been predicted to be rated for the target user.

### Determination of the parameter *μ*

Before evaluating the results of the model proposed in this study (STUBCF), the weighting factor *μ* for social and trust relationships needs to be discussed and its optimal value needs to be determined. The CF technique framework is employed in this study to ascertain the value of *μ*:

Step 1: The dataset is randomly split into 80% training and 20% test sets during the experimental phase.

Step 2: The spectral clustering algorithm groups users according to their similarity value Sim.

Step 3: Using the nearest neighbor score prediction approach, locate the target user’s closest neighbor users in the cluster where the target user belongs to and compute the predicted score.

Step 4: Evaluate the results using Mean Absolute Error (MAE) evaluation metric.

**Fig 2:**
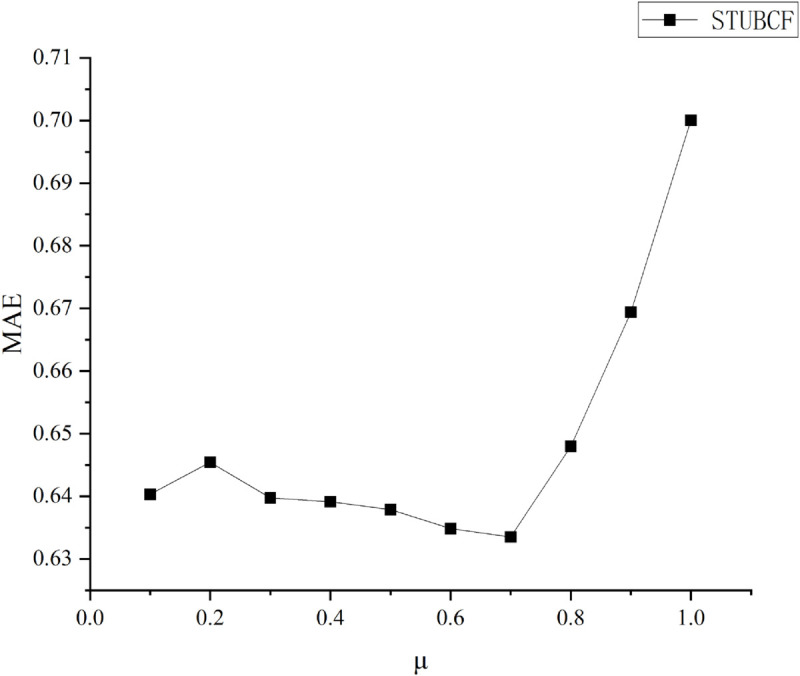
MAE values corresponding to different weighting factors. MAE values corresponding to different weighting factors.

The corresponding MAE values when *μ* takes values between 0 and 1 are compared in Fig 2. In this figure, it is shown that the result of MAE is minimized when *μ* takes the value of 0.7, which indicates that the recommended results are more accurate. Therefore, in this study, μ=0.7 is chosen as the value to be taken for the following comparison experiments.

In real life, people prefer to socialize with people they trust, which validates our hypothesis that people with high levels of trust have closer relationships with each other. This finding is consistent with reality.

### Contrast model

Several traditional algorithms have been selected for this study and the algorithm designed for this study, which consists of social relationship, trust relationship and spectral clustering algorithms, has also been considered. In this study, separate experiments were conducted on each component of the traditional algorithms and its own algorithm and their MAE values in terms of prediction accuracy were compared. Through these experiments, this study aims to evaluate the performance of the different algorithms and to determine whether the algorithm designed in this study can achieve better results in terms of prediction accuracy. Such a comparison will help to reveal the contribution of each component in its own algorithm and provide guidance for further improvement of the algorithm. The traditional algorithms include User-Based Collaborative Filtering Recommendation Algorithm (UBCF) [1] and Collaborative User Clustering Modeling Based Information Filtering (UCMF) [9].

The specific comparison of various recommendation algorithms is as follows:

1. User-based collaborative filtering recommendation algorithm (UBCF): this algorithm predicts ratings or preferences for items based on users’ similarities. UBCF has the advantage of being easy to implement and interpret, and it can make recommendations based on a user’s historical behavior. However, the disadvantage is that it needs to compute user similarity, which requires a lot of computational resources, and may recommend poorly for new users or cold-start problems.
2. Information Filtering via Collaborative User Clustering Modeling (UCMF): this study is based on matrix decomposition, which uses the interaction between users and product labels to cluster users. However, the method does not delve into user features, product features or rating information, and is insufficient for mining user behavior patterns. In order to conduct comparative experiments, this study adjusts the number of clustering clusters for UCMF.
3. K-means based user clustering collaborative filtering recommendation (K_UBCF): this study aims at CF recommendation based on user clustering, clustering users with the help of the k-means method, and improving the UBCF algorithm to conduct comparative experiments.
4. CF recommendation based on social relationship-based user clustering (SUBCF).
5. User clustering CF recommendation based on trust relationship (TUBCF).

In this study, we focus on social and trust relationships, and the results show that among user relationships, trust relationships account for a greater proportion. In order to deeply investigate the degree of influence of social relationship and trust relationship on the results, this study conducts corresponding experiments on the above two algorithms, SUBCF and TUBCF, for comparative validation.

### Comparative experiments and analysis of results

In the experimental phase of this study, the MovieLens 100K real dataset is split into a training set and a test set, with the training set making up 80% and the test set 20%. The proposed algorithm is used to simulate the actual recommendation situation of users. The following are the experimental results and analysis.

1. Changes in MAE with different number of clusters

**Fig 3:**
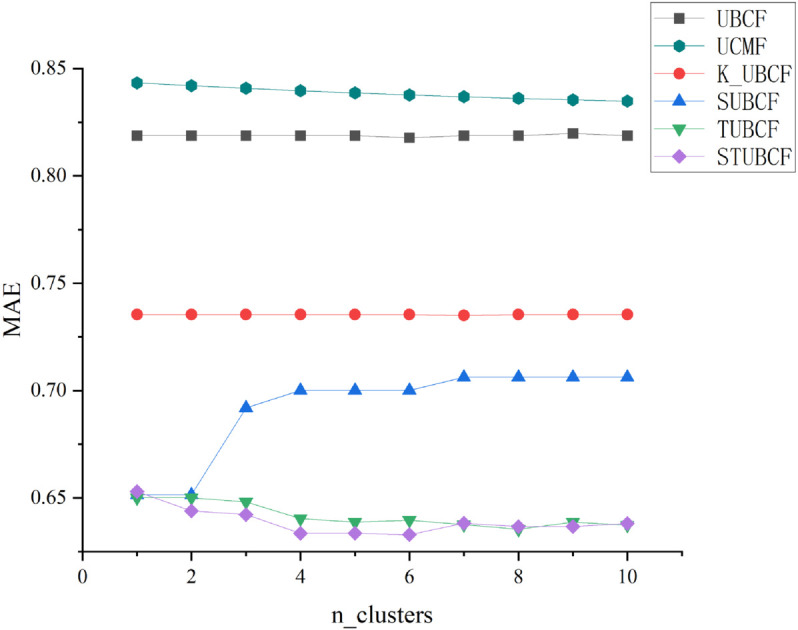
Changes in MAE with different number of clusters. Changes in MAE with different number of clusters.

As shown in Fig 3, the number of clusters has an effect on the algorithm’s recommendation accuracy during the user clustering process. According to the results of the previous experiment, the weight factor is set to μ=0.7, and also the number of nearest neighbor users k_neighbors is set to 10.

Given the results in Fig 3, the following conclusions can be made:

(1) The traditional user-based collaborative filtering algorithm (UBCF) does not cluster users, thus resulting in the Mean Absolute Error (MAE) value remaining stable and high. The user-based clustering CF algorithm (UCMF), due to the lack of in-depth mining of the relationship between users and the high sparsity of the data, is unable to avoid a significant prediction error even though the MAE value decreases with an increase in the number of clustered clusters. On the basis of UBCF, the CF recommendation based on k-means algorithm (K_UBCF) is used, while the MAE value does not vary significantly as the number of clustering clusters increases, it is evident that clustering users can improve prediction accuracy;

(2) The effect of SUBCF, TUBCF, and STUBCF is better than the other three algorithms.

In Fig 3, the MAE value of SUBCF is significantly higher than those of TUBCF and STUBCF. This occurs because SUBCF performs user clustering based solely on social relationships. When the number of clusters increases, over-segmentation of user groups may lead to two key issues: 1) Degradation of neighbor quality: Social relationships measure the breadth of interest associations but not the depth of interest associations. Over-clustering fragments users into subgroups where neighbors may share strong social ties but diverge in interest score preferences. This introduces noisy references, elevating MAE value; 2) Amplification of data sparsity: Social relationship-driven clustering may assign users to smaller subgroups. The historical ratings of users within subgroups are already sparse, and excessive segmentation further limits the diversity of available reference data, exacerbating the upward trend of MAE value.

The MAE values of TUBCF and STUBCF fluctuate slightly, and with the increase in the number of clustered clusters, there is slight overlap between the MAE values of TUBCF and STUBCF, which further proves the importance of the trust relationship. Among all the compared algorithms, the prediction results of STUBCF are always the best;

(3) The MAE value does not exhibit a declining trend as the number of clustering clusters increases, suggesting that the number of divided groups is not the more the better. Because CF recommendation is implemented within the group where the user is located, too fine user clustering division may lead to valuable reference items lost in the outside of the cluster. Therefore, choosing the ideal number of clustering clusters is essential to enhancing the recommender system’s performance.

2. Changes in MAE when the number of nearest neighbor users increases

**Fig 4:**
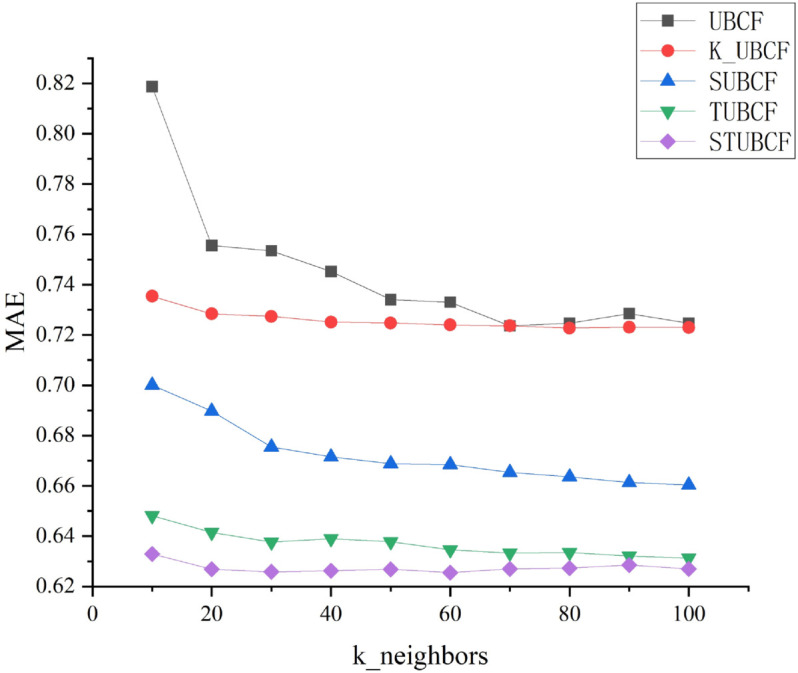
Changes in MAE when the number of nearest neighbor users increases. Changes in MAE when the number of nearest neighbor users increases.

Fig 4 shows how the quantity of nearest neighbor users affects the accuracy of recommendation outcomes when selecting recommendation neighbors for the algorithm presented in this study. In the experiments, the weight factor is set to μ=0.7 and the number of clusters is set to 6. Since UCMF is not based on the nearest neighbor prediction method to compute the prediction scores, this part of the experiment is not carried out for UCMF in this study.

According to Fig 4, the following conclusions can be drawn:

(1) With the increase of nearest neighbor users (k_neighbors), the MAE values of the other four algorithms, except for the SUBCF algorithm, will first decrease and then stabilize, but when k_neighbors increases to a certain degree, the MAE value will slightly increase. This indicates that as k_neighbors increases, the amount of reference information used for prediction scoring also increases, however, to a certain extent, some of the neighbors’ reference information may become less important, and may even interfere with the prediction results to a certain extent, leading to an increase in the error. It is worth noting, however, that SUBCF decreases the MAE value significantly with the increase of k_neighbors, which indicates that it is meaningful for the SUBCF algorithm to increase the reference information of the nearest neighbor users.

(2) Compared with SUBCF, TUBCF has higher recommendation accuracy, which confirms the idea that trust relationship is more important compared to social relationship. Among these five compared algorithms, STUBCF consistently has the lowest MAE value, which suggests that the study’s suggested algorithm is more accurate in making recommendations and is more competitive. At the same time, it also shows that social relationships and trust relationships are complementary and indispensable in social networks.

## Conclusion

This study proposes a user clustering collaborative filtering recommendation algorithm (STUBCF) that incorporates implicit social relationships and trust relationships. This study incorporates the following aspect to address the data sparsity and cold-start issues: first, the Jaccard similarity coefficient is utilized to quantify the strength of the social relationship between users, but since this method ignores the size of the rating values, this study introduces the trust relationship. By analyzing the intersection of other users’ goods in the target user’s ratings with the size of the ratings, the trust relationship between users is established, so that the user similarity is calculated by linearly combining the social relationship and the trust relationship, which effectively mitigates the data sparsity and cold-start problems. Secondly, the spectral clustering algorithm can effectively cluster and classify users in their social relationships. Therefore, this study adopts the spectral clustering algorithm to cluster and divide users, and finally applies the user-based CF recommendation method within the cluster where the target user is located, which improves the accuracy of recommendation. The efficacy of the model was tested using the MovieLens 100K dataset, and the findings indicate that STUBCF performs better than alternative techniques.

In future research, we intend to collaborate with data institutions to obtain richer and more diverse datasets, including both sparse and dense ones. By comparing and analyzing the performance of our model under different data density scenarios, we aim to further refine and generalize the validation framework, thereby enhancing its rigor and applicability. Additionally, this study has not yet considered the influence of user attribute data on user similarity. Future work will explore how user attributes can impact user similarity, with the goal of improving the accuracy and robustness of recommendation systems.

## Supporting information

S1 File The values used to build graphs. (ZIP)
